# Editorial: Human biomonitoring (HBM) as a tool to support policy and regulatory action to prevent chemicals exposure

**DOI:** 10.3389/ftox.2024.1376890

**Published:** 2024-03-15

**Authors:** Susana Viegas, Carla Martins, Ricardo Assunção

**Affiliations:** ^1^NOVA National School of Public Health, Public Health Research Centre, Comprehensive Health Research Center (CHRC), NOVA University Lisbon, Lisbon, Portugal; ^2^ Egas Moniz Center for Interdisciplinary Research, Egas Moniz School of Health and Science, Caparica, Portugal; ^3^ Centre for Environmental and Marine Studies (CESAM), University of Aveiro, Aveiro, Portugal

**Keywords:** human biomonitoring, exposure assessment, risk assessment, science to policy, chemical safety

Human biomonitoring (HBM) is a powerful tool, offering insights into the internal exposure to various chemicals at both individual and community levels. The data from HBM not only inform regulatory decisions but also provide evidence that can further support the prioritization of actions and policy measures. Furthermore, HBM can also provide awareness of the time trends of exposure, identify vulnerable groups, assess the effectiveness of existing regulatory measures and promote more comprehensive and accurate health impact assessments. Past and ongoing research projects have convincingly demonstrated the utility of HBM across diverse regulatory frameworks, solidifying its role in the science-to-policy interface.

In this context, some research groups have carried out studies utilizing HBM to evaluate programs aimed at preventing chemical exposure in workplaces. This is the case of the paper of Peralta et al. where it was investigated the blood lead levels of a sample of potters and analysed the association with the type of glaze used in the scope of the approved Pottery Program that aims to promote lead-free pottery production (Peralta et al.). The use of HBM conclusively demonstrated that adopting lead-free glaze in the production of pottery was associated with lower blood lead levels in potters (Peralta et al.).

Morton et al. explored occupational exposure trends using HBM data collected over more than 20 years in Great Britain (GB). The data was stored within the Health and Safety Executive (HSE) database, which holds more than 950,000 results from 120,000 workers in 8,000 companies. The data were collated for all biological monitoring results for lead, Mercury, benzene, and hexamethylene diisocyanate exposures where there have been some regulatory drivers within the reported time of the data searched. The HBM data allowed observing strong evidence of reductions in the exposure of GB workers to all the chemicals, likely attributable to the impact of national, regional and global regulatory actions. However, the study also highlights the loss of high-exposure industries and the increase in automation or substitution as potential determinants. Moreover, it allowed to conclude that exposures are dynamic between sectors over time and sectors such as waste and recycling (lead, Mercury) and tunnelling through contaminated land (benzene) are sectors or tasks of increasing concern (Morton et al.).

Rodriguez-Carrillo et al. present a compelling narrative review summarizing recent findings focusing on the role of brain-derived neurotrophic factor (BDNF) as a biomarker of effect for neurodevelopmental alterations during adolescence, based on the health effects of exposure to environmental chemical pollutants. Derived from three pilot studies developed within the HBM4EU project, the findings suggest that exposure to various chemical pollutants such as fine particle matter, perfluoroalkyl compounds, heavy metals, bisphenols, and nonpersistent pesticides may alter circulating BDNF levels in healthy populations. Therefore, BDNF could be used as a valuable effect biomarker for investigating the developmental neurotoxicity of some chemical pollutants (Rodriguez-Carrillo et al.).

In the shipbuilding industry, Pigini et al. conducted a study aiming at evaluating the oxidative stress effects due to occupational exposure to styrene and other chemicals. Using biomarkers of exposure (for styrene) and effect (oxidative stress), the authors concluded that the workers performing the tasks of painting are the most exposed to styrene and show higher concentrations of 8-oxo-7,8-dihydroguanosine (8-oxoGuo), one of the biomarkers of effect. Workers performing the tasks of wood refining and welding demonstrated reduced levels of exposure to styrene but higher concentrations of 8-oxoGua and 8-oxodGuo, likely linked to exposure to other chemicals (Pigini et al.).

Lastly, a paper developed by the Europe Regional Chapter of the International Society of Exposure Science’ (ISES Europe) HBM working group (ISES Europe HBM WG) proposed the development of a FAIR Environment and health registry (FAIREHR) (Zare Jeddi et al.). This paper envisions a registry that facilitates preregistration of studies in exposure sciences and environmental epidemiology using HBM (as a starting point), across all areas of environmental and occupational health globally, with a dedicated web-based interface, to be electronically searchable and to be available to all relevant data providers, users and stakeholders. The authors advocate for the implementation of FAIREHR since it is expected to yield significant benefits in terms of enabling more effective utilization of HBM data (Zare Jeddi et al.).

Considering the impactful findings presented in these papers, reinforcing the utility of HBM, there is an evident call for continued investment in biomonitoring programs. Such programs are crucial for generating representative HBM data, covering relevant population groups, and enabling more accurate risk assessments and robust policies. The establishment of comprehensive, accessible database platforms was highlighted as key to promoting the integration of HBM data into risk assessment studies and regulatory actions.

